# Infantile epileptic spasms syndrome: Mechanisms and therapeutic approaches

**DOI:** 10.1016/j.neurot.2025.e00822

**Published:** 2025-12-18

**Authors:** Carl E. Stafstrom

**Affiliations:** Division of Pediatric Neurology, Department of Neurology, Johns Hopkins University School of Medicine, Baltimore, MD, USA

**Keywords:** Infantile epileptic spasms syndrome, Hypsarrhythmia, Electrodecrement, Animal models, Adrenocorticotropic hormone, Corticosteroids

## Abstract

Infantile epileptic spasms syndrome (IESS) is a developmental and. epileptic encephalopathy with unique clinical and electrographic features, including seizure semiology (spasms), numerous and diverse etiologies spanning structural, genetic and metabolic causes, characteristic interictal (hypsarrhythmia) and ictal (electrodecrement) electroencephalogram (EEG) patterns, and responsiveness to “standard” pharmacological therapies (adrenocorticotrophic hormone, high-dose corticosteroids, vigabatrin) that are not commonly used in other epilepsy syndromes. Despite these long-recognized clinical features and laboratory investigations using a multiplicity of animal models with different epileptogenic mechanisms, the neurobiological underpinnings of IESS remain poorly understood, hampering the development of alternative treatments. This commentary discusses three aspects of IESS intended to raise fundamental clinical and mechanistic issues to afford greater understanding of the syndrome – nomenclature, EEG findings, and selected emerging animal models that might shed light on IESS pathophysiology and guide therapy development.

## Introduction

Infantile Epileptic Spasms Syndrome (IESS) is a developmental and epileptic encephalopathy (DEE) with numerous unique features. Despite its clinical description in 1841 and characterization of its electroencephalographic (EEG) correlates and syndromic nature in 1952, current treatment regimens have remained largely unchanged for several decades [1]. Available “standard” pharmacological treatment regimens - adrenocorticotropic hormone (ACTH), high-dose oral corticosteroids (OCS), and vigabatrin (VGB) (especially but not exclusively for patients with tuberous sclerosis complex) - are suboptimal with regard to their effectiveness and can be rife with side effects. Moreover, the neurobiological mechanisms underlying IESS remain largely unknown. The observation that numerous etiologies can disrupt neurodevelopmental pathways and cause IESS [2] presents a hindrance to a unified pathophysiological understanding, and animal models, though promising, have not yet provided the hoped-for clarity. This invited commentary provides an opportunity to consider a few selected recent advances and potential controversies about IESS. My aim is to generate discussion of some of the fundamental knowledge gaps of IESS. I focus on three topics: (1) revisions in the nomenclature and classification of the disorder, (2) issues pertaining to the interictal EEG pattern – hypsarrhythmia - including hypotheses about its underlying pathophysiology, and (3) the status of preclinical models in elucidating the neurobiology and pointing toward novel therapeutic approaches. This review does not attempt to discuss all aspects of IESS, as comprehensive reviews are available [[3], [4], [5], [6], [7]]. As an overview, the clinical features of IESS are summarized in Table 1.

**Table 1:** Infantile epileptic spasms syndrome.

Age of onset (peak)	1–24 months (6 months)
Seizure semiology	Flexion or extension spasms
Etiologies	Numerous, spanning genetic and acquired causes (structural, genetic, infectious, metabolic, unknown)
Associated signs and symptoms	Cognitive-developmental plateau or regression
EEG features	Interictal: hypsarrhythmia (not all cases) Ictal: Electrodecrement
Treatment responsiveness	Corticosteroids (prednisolone), adrenocorticotrophic hormone (ACTH), vigabatrin
Prognosis	Poor: Subsequent cognitive impairments and predisposition to other seizure types, dependent on specific etiology

## Nomenclature

It is important to discuss nomenclature to ensure clear communication among epilepsy specialists, other health care professionals, and caregivers, as well as to emphasize a direct relationship to treatment decisions and research initiatives. The nomenclature around spasms with an epileptic basis (epileptic spasms or ES) has evolved over many years [8]. For example, infantile spasms (IS) is the subset of ES occurring during the first year or two of life. Authors have ascribed different criteria when defining IS, sometimes leading to confusion about what population is being studied [8].

With the 2022 publication of a position statement by the International League Against Epilepsy (ILAE), a “new” disorder was defined - Infantile Epileptic Spasms Syndrome IESS [9] (Table 2). The disorder is not actually new, of course, but its renaming recognizes that IESS is an epilepsy syndrome, whereas previous terms such as infantile spasms semantically encompass an age range (infantile) and a seizure type (spasms) without requiring additional features of a syndrome. The three mandatory features of IESS in the ILAE definition are: (1) epileptic spasms as the seizure semiology, (2) onset between 1 and 24 months of age (peak, 3–12 months), and (3) an epileptiform EEG. Notably, the 2022 IESS definition does not require hypsarrhythmia on EEG or developmental arrest, and ES as a seizure type can occur without concomitant hypsarrhythmia or developmental delay, arrest, or regression.

**Table 2:** Terminology.

TERM	DEFINITION
Infantile spasms (IS)	Seizure with spasm semiology, <2 years old
Epileptic spasms (ES)	Seizure with spasm semiology, any age
Infantile epileptic spasms syndrome (IESS)	Syndrome including spasms with epileptiform EEG, with or without hypsarrhythmia and with or without neurodevelopmental plateau/regression
West syndrome (WS)	Triad of IS, hypsarrhythmia, and neurodevelopmental plateau/regression (subtype of IESS)

ES is a broader term that covers the clinical spasm semiology at any age, though most cases of IESS occur under two years of age. Therefore, the term IESS is now used to refer to ES with an epileptiform EEG but not necessarily hypsarrhythmia in children within the infantile age range with or without developmental stagnation. In the past, there was lack of consensus as to whether the inclusion of hypsarrhythmia or developmental delay are required to begin therapy and whether prognosis is different if hypsarrhythmia or developmental deficits are not present. It has been argued that these additional “syndrome” features should not preclude or delay treatment in a child with infantile spasms [8,9]. The recent designation of IESS as a syndrome clarifies its epileptic basis and emphasizes the high risk for epileptic and cognitive sequelae that require close monitoring. Hopefully, the IESS assignation will allow for more consistent and precise disease classification for clinical and research purposes (Table 2).

Infantile spasms was first described in 1841 by Dr. William West, a British physician whose 4-month-old son developed episodes of “bobbings of the head forward” that increased in frequency and severity, eventually including truncal flexions and limb flexions/extensions, accompanied by developmental regression. In an impassioned letter to Lancet, Dr. West sought advice from the wider medical community on how to treat his son's condition [10]. In recognition of Dr. West's observations, the disorder was later designated “West syndrome” by Gastaut at a 1960 meeting about infantile myoclonic epilepsies held in Marseilles, France [[11], [12], [13]]. The term West syndrome has undergone numerous reinventions over time. Gibbs and Gibbs identified the unique interictal EEG pattern that often accompanies IS, which they called hypsarrhythmia [14]. Dulac and colleagues suggested that West syndrome was a subset of IS when hypsarrhythmia was present [15]. The West Delphi consensus group proposed the term Infantile Spasms Syndrome as entailing spasms and hypsarrhythmia but did not require regression [16]. “Epileptic” was added to the current syndrome name to differentiate IESS from its mimics and emphasize its epileptic nature [9].

In modern parlance, West syndrome comprises the triad of epileptic spasms (ES), hypsarrhythmia on interictal EEG, and neurodevelopmental regression. However, since IS can also occur without hypsarrhythmia or regression, the criteria defining West syndrome may be overly restrictive. Regarding age of onset, historically, IS has referred to the first year (or two) of life, whereas ES can occur at any age. To lessen potential confusion, some authors discourage the use of the eponym West syndrome [5]. It is my view that the term West syndrome can be retained as a subset of IESS, acknowledging its important historical context.

IESS is classified as a DEE, in which the seizures or accompanying EEG abnormalities cause or exacerbate the patient's cognitive deficits (encephalopathy) [17]. DEEs exact an immense toll on patients, families, the health care system, and society at large [18,19]. Affected individuals suffer from ongoing seizures and cognitive dysfunction despite extensive pharmacological treatment. Therefore, novel therapies are needed, a situation that is reliant upon clarifying the pathophysiology through pre-clinical models (see following sections).

Historically, around 60 % of IESS cases have been attributed to a structural, metabolic, or infectious cause and the remaining 40 % were considered to have a genetic variant or unknown cause [6,20,21]. However, this ratio is changing due to advances in diagnostic genetic and imaging modalities. Numerous genes have been linked to IESS, encompassing those encoding ion channels, various aspects of synaptic function, metabolic regulation, and protein production or modification, among others [6,21,22]. An incredibly diverse spectrum of molecular and cellular mechanisms converges onto a relatively stereotyped clinical presentation. This genetic heterogeneity makes a simple targeted treatment approach based on etiology quite challenging, as discussed below under Mechanisms. Ideally, identification of common downstream pathways signifies network dysfunction that might be informative across etiologies.

## EEG features – hypsarrhythmia and electrodecrement

The most common EEG patterns accompanying IESS are interictal hypsarrhythmia and ictal electrodecrement (during a spasm, the EEG shows a large-amplitude slow wave followed by diffuse voltage attenuation with reduction of all voltages for several seconds). Several aspects of these EEG patterns warrant discussion: their description and variants, challenges and opportunities in diagnosing them more accurately, their pathophysiological and cellular basis, and their prognostic implications.

### Hypsarrhythmia and its diagnosis

Hypsarrhythmia is a unique electrographic pattern whose underlying cellular and network basis is essentially a complete mystery. Early work proposed that hypsarrhythmia reflected aberrant firing within cortico-thalamic pathways, requiring involvement of both cortical and subcortical structures, but the details of hypsarrhythmia and spasms physiology, including generation site, cellular and subcellular signaling pathways, and termination targets remain to be determined [[23], [24], [25], [26]].

The interictal hypsarrhythmia EEG pattern was described and named more than 70 years ago by Gibbs and Gibbs as consisting of high-amplitude (>200 μV) irregular slow waves (in the delta range, <3 Hz) with intermixed multifocal sharp waves and spikes, with little uniformity of waveforms from channel to channel on the EEG [14]. Therefore, this pattern has been referred to as “chaotic.” In Greek, “hyps” means “mountainous” (high amplitude), “a” means not, and “rhythm” means rhythm. Due to its chaotic appearance, hypsarrhythmia has been hypothesized to reflect neuronal network dysfunction leading to the encephalopathy inherent in IESS. Actually, hypsarrhythmia is not present initially on the EEG of ∼20–40 % of children who present with IS [27]. Sometimes hypsarrhythmia begins weeks or more after than the clinical spasms, and sometimes hypsarrhythmia never occurs [28]. Moreover, the EEG pattern may reveal a “modified” form of hypsarrhythmia without the full criteria being met. As strictly described by Hrachovy and colleagues, examples of modified hypsarrhythmia variants include hypsarrhythmia confined to one hemisphere (hemihypsarrhythmia), hypsarrhythmia with a single interictal spike focus embedded in the EEG background, hypsarrhythmia that resembles suppression-burst, and hypsarrhythmia with slowing but few or no spikes [28]. These subtle or “modified” variants could lead to an alternative prognosis or management considerations [29], and some authors suggest that the term be abandoned [30].

Hypsarrhythmia is a qualitative term, so it is uncertain what is implicit in its characterization as electrophysiological “chaos” Is this pattern truly disorganized or are there aspects of organization or synchrony hidden within the waveforms that could provide information about the brain's pathophysiology? Some newer quantitative investigations into the underlying basis of hypsarrhythmia, using advanced signal analysis techniques, reveal stronger functional connections among neuronal networks in patients with spasms than in controls [31,32]. In a preliminary report on three children with IS, a measure called the phase synchronization index was used to evaluate the hypothesis that waveform phases during hypsarrhythmia were less synchronized than during the ictal electrodecrement [33]. The authors found the opposite – there was more prominent synchrony of waveforms during hypsarrhythmia than during the electrodecrement, suggesting that there could be some underrecognized organization in the network firing during hypsarrhythmia than could be detected visually. This exploratory study does not address either the underlying cellular basis of the neuronal activity during hypsarrhythmia or electrodecrement, and it does not address the concern that hypsarrhythmia is responsible for developmental deficits. Indeed, the cellular physiology of hypsarrhythmia remains a critical knowledge gap - what exactly is happening in cortical neurons and networks during hypsarrhythmia, on the synapse, ion channel, and network connectivity levels? Little experimental attention has been paid to this topic; the EEG findings of IESS are very difficult to model and study, perhaps reflecting phenomena limited to humans. Promising studies using animal models are discussed below, though, for the most part, available EEG recordings in animal models of ES do not convincingly recapitulate the human EEG; side-by-side EEG recordings from various animal models demonstrate wide variability and imperfect similarity with human data [34]. An exception is the TTX model, in which the EEG does bear a reasonable semblance to human hypsarrhythmia and electrodecrement [35]).

The subtle manifestations and modified forms can make hypsarrhythmia difficult to diagnose, even for the experienced electroencephalographer, and the poor interrater reliability for diagnosing hypsarrhythmia has been amply documented [36]. Recent work of Mytinger and colleagues [37] strives to develop a reliable interictal EEG scoring system for IESS, having a high level of interrater reliability and offering a promising scoring method to guide clinical studies, independent of the need to grade hypsarrhythmia. Their method, called the Burden of AmplitudeS and Epileptiform Discharges (BASED), quantifies a 0- to 5-point score based on the amplitude of the EEG background, location of epileptic discharges, and number of spike foci within defined EEG epochs [37]. This method is associated with a good interrater reliability (ĸ = 0.86), allows comparison of EEG findings before vs after treatments, and provides a tool for EEG rating to enhance clinical and research studies [38]. Other future approaches might entail artificial intelligence (AI) methods to identify spasms clinically, by “teaching” AI algorithms to recognize the overt and subtle clinical or EEG manifestations of ES [39]. This approach could be especially useful in resource limited areas.

Children with IESS may have no hypsarrhythmia, hypsarrhythmia mainly during sleep, or hypsarrhythmia or its modified forms during both sleep and wakefulness. If hypsarrhythmia is present, it is likely that the child spends considerably more time in the hypsarrhythmia state, compared to the proportion of time during which spasms occur. Based on evidence, the therapeutic goal in IESS is to eliminate both the interictal hypsarrhythmia (when present) and the ictal spasms [4]. This goal drives the inclusion/exclusion criteria of clinical trials and clinical care. It makes sense that resolution of hypsarrhythmia is a worthwhile target of therapy - the presence of hypsarrhythmia throughout the majority of the day and night in a child with IESS may account for the developmental plateau or regression often seen, though this remains speculative. It makes intuitive sense that the chaotic EEG pattern of hypsarrhythmia represents disruption of the brain's normal network function, while the actual seizures (spasms) occupy at most several minutes over the course of a day. But some children with ES do not exhibit hypsarrhythmia, especially if the EEG is done shortly after the spasms begin, but there is no data to support withholding standard therapy in a child with ES who lacks hypsarrhythmia [8,40].

A final comment about IESS and other epileptic encephalopathies regards labeling them as “catastrophic” or “devastating” [41,42]. In my opinion, these judgmental adjectives serve no useful purpose, and in fact, can cause unnecessary despair among family members. Instead, IESS might realistically be regarded as a “high risk” epilepsy syndrome that often - but not always - carries a poor prognosis for epilepsy and development. While varying considerably by etiology, promptness of diagnosis and treatment, and other factors, more than half of children with IESS achieve spasms control and up to a quarter have normal or near normal cognition (IQ > 68) [[43], [44], [45]] With time, expanded funding, and concerted, targeted research efforts working toward viable, more effective novel therapies, I am confident that we can move the needle away from catastrophic to hopeful [46].

### Pathophysiology of hypsarrhythmia and electrodecrement

While the neurobiological basis of the primary EEG patterns in IESS - interictal hypsarrhythmia and ictal electrodecrement - remain unknown, there have been recent attempts to determine their pathophysiology. This knowledge gap, in part, relates to species differences (rodents and other animal models do not fully approximate the complexity of the human brain, and the species differences are reflected in disparate EEG patterns) and, in part, to the inherently complicated challenge of trying to sort out cellular correlates from scalp-recorded electrographic patterns.

**Fig. 1:**
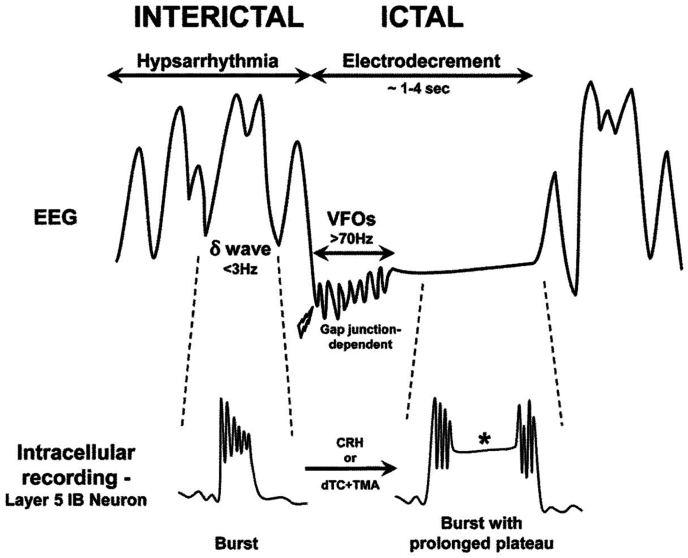
Schematic showing characteristic electroencephalogram (EEG) findings in infantile epileptic spasms syndrome. Top: Hypsarrhythmia (interictal), if present, consists of chaotic, high-voltage irregular low waves (delta range, <3 Hz) with superimposed multifocal sharp waves and spikes. Lightning bolt indicates onset of a clinical spasm. The EEG sometimes exhibits very high frequency oscillations (VFOs, >70 Hz) that are mediated by gap junctions, followed by electrodecrement (attenuation of voltage during the clinical spasm). Once the spasm ends, the electrodecrement ceases and hypsarrhythmia resumes. Bottom: Electrophysiological cellular correlates to the various phases of the EEG are indicated. During hypsarrhythmia, layer 5 pyramidal neurons (intrinsic bursting (IB) type) fire in bursts, accompanying each EEG delta wave. These bursts require N-methyl-d-aspartate (NMDA) receptors and GABA_B_ receptors. During electrodecrement, delta waves are suppressed and layer 5 IB neurons are further depolarized, leading to prolonged plateau potentials (asterisk). These plateaus are maximized by an intracellular alkaline pH and involve glutamate release and increased Ca influx. The transition from interictal to ictal firing can be induced experimentally by the endogenous proconvulsant corticotropin-releasing hormone (CRH) or exposure to the combination of d-tubocurarine (dTC, an acetylcholine receptor antagonist) and trimethylamine (TMA), an alkalizing agent that enhances gap junction opening). Though simplified, this scheme illustrates potential targets for novel therapeutics (see text). Source: Reprinted from Ref. [69].

Traub and colleagues proposed plausible, experimentally testable hypotheses for hypsarrhythmia and electrodecrement based on experimental and modeling data [47]. The cellular basis of the irregular slow waves that characterize hypsarrhythmia was hypothesized to reflect activity of a subtype of neocortical layer 5 neurons called intrinsic bursters (IBs) [48]. IBs are the source of normal cortical delta waves, such as those seen during deep sleep. A hypsarrhythmia-like firing pattern in IBs can be produced in neocortical slices by alkalinizing their intracellular milieu with an agent such as trimethylamine (TMA), which reduces the activity of early-developing interneurons by blocking acetylcholine receptors. This results in disinhibition of IBs, enhancement of glutamate release, and markedly increased magnitudes of both IB neuron bursting and delta rhythms, leading to a hypsarrhythmia-like pattern - each burst in layer 5 IB neurons corresponds to a delta wave recorded on surface EEG or an abnormal slow event in the IS model (Fig. 1).

On the other hand, electrodecrement is thought to occur when a sufficient number of layer 5 IBs develop sustained plateau depolarizations (Fig. 1) [49]. These plateaus require intact calcium (Ca) conductances and an alkaline cytosolic pH, as with TMA exposure, which has multiple effects on neurons including enhancement of Ca conductances, increased glutamate release, opening of gap junction channels, and blockage of K conductances [39]. Excessive Ca influx into IB neurons could be excitotoxic and contribute to the encephalopathy in IESS. The requirement of an alkaline intracellular pH is intriguing, in that drugs that are mildly acidic, such as acetazolamide and topiramate, as well as high-dose OCS and the ketogenic diet, are sometimes successful in suppressing ES [[50], [51], [52]]. In the case of the ketogenic diet, numerous potential mechanisms involved in pH regulation could predispose neurons to acidosis, including acid sensing ion channels, gap junctions, and effects of acidosis on other ion channels and synaptic function [53].

These observations also raise the possibility that Ca conductance blockers could be anticonvulsant or even neuroprotective in IESS. Of note, corticotrophin releasing hormone (CRH), a potent convulsant in early brain development [54], increases Ca conductance and addition of CRH to neocortical slices results in facilitated burst discharges and plateau potentials [55]. Therefore, CRH facilitates neuronal plateaus, further supporting the hypothesis that CRH receptor blockade or Ca channel blockade (or both) might be therapeutic in IESS. Several currently available agents or metabolic therapies down regulate CRH receptors (ACTH [54]) or block Ca channels (e.g., verapamil [56] - although verapamil has a variable and limited effectiveness as an antiseizure drug; and fructose-1,6-diphosphate, which links excitability with metabolic regulation [57]). Ca channel blockers seem ripe for investigation in IESS models.

Very fast EEG oscillations (VFOs, >70 Hz) are sometimes seen during the early part of the ictal electrodecrement (Fig. 1). Intracellular alkalinization enhances VFOs, which likely reflect activity in neurons in more superficial cortical layers (e.g., layers 2 and 3), widely transmitted by gap junctions between cortical pyramidal neurons, as these oscillations are blocked by gap junction inhibitors [58,59]. Gap junctions represent an attractive mechanism for the rapid activation of neuronal networks and they are especially prominent early in development [[60], [61], [62]]. The use of gap junction inhibitors in IESS has not yet been reported.

It might be envisioned that ES-associated EEG changes result from an entire cortical network of bursting pyramidal cells, intermittently interrupted by brief suppressions of these bursts by an alkaline intracellular pH and other factors, producing the ictal component. The transition from baseline hypsarrhythmia to ictal electrodecrement and then back to hypsarrhythmia remains unexplained by existing data, but the intriguing ideas described here allow several pathophysiological hypotheses to be assessed.

## Treatment

Details about IESS treatment are beyond the scope of this commentary, but I include a few perspectives as a prelude to the subsequent section on mechanisms and novel therapies. Numerous treatment algorithms and protocols have been proposed and investigated, reviewed elegantly by Hussain [4].

The first successful treatment option of IESS was adrenocorticotrophic hormone (ACTH) [63]. Since then, numerous reports have attempted to determine the optimal formulation, dose, treatment duration, and options if spasms recur [4,64]. More recently, the OCS prednisolone has been offered as a cost-effective alternative to ACTH [65]. Together, ACTH and OCS are termed “hormonal” therapies. ACTH and OCS are similarly efficacious [66]; ACTH may capture some OCS non-responders [67]. Meta-analysis and case series have shown that high-dose prednisolone is not inferior to ACTH [[68], [69], [70]]. Hormonal therapies are typically used as first line treatment for IESS [66,71] but they can carry significant potential side effects (hypertension, cushingoid appearance, immune suppression) that limit their long-term use. The mechanisms by which hormonal therapies suppress ES are unknown [72].

The third member of the first line/standard therapy group is VGB, a ɤ-amino-butyric acid (GABA) transaminase inhibitor that has been found in several studies to be especially effective for one common etiology of IESS – tuberous sclerosis complex [73]. Some authors favor using VGB as an initial choice in non-TSC cases, even prior to hormonal therapies, but the reported effectiveness varies considerably and the optimal therapy duration has not been determined [74]. If high-dose corticosteroids fail to suppress ES, there is no difference between the effectiveness of ACTH vs VGB as the next treatment [75]. VGB has been associated with irreversible visual field defects and MRI signal changes, especially in subcortical areas, which has limited the enthusiasm for this drug. However, VGB-Associated Brain Abnormalities on MRI (VABAM) are often transitory [76], and retinal toxicity is less frequent in young children [77]. In summary, hormonal therapies are most often selected prior to VGB [78,79]. There is some geographical preference in clinical use of the various treatment options; VGB is used as first line therapy more often in Europe [61].

There is increasing interest among clinicians to begin combination therapy with both a hormonal agent and VGB at the start of IESS treatment [4]. This approach carries the benefit of addressing multiple mechanisms at the outset rather than delaying the second agent, aiming to reduce the spasms and hypsarrhythmia in a more expeditious fashion. There is some rationale for combination therapy, including rapid sequential therapy which has been shown to achieve a 3-month remission rate of ∼75 % [78] but there is not yet a standardized protocol. However, in the International Collaborative Infantile Spasms Study (ICISS), there appeared to be early (up to 6 weeks) benefit from the combination of hormone plus VGB as initial therapy compared with hormone alone on electroclinical response [80]; however, there was no difference in developmental or seizure outcomes at 18 months [81]. Of course, the cost and side effect burden are increased with combination therapy.

Non-standard approaches include benzodiazepines, valproic acid, topiramate, cannabidiol, ketogenic diet, surgery – none of these targets a single specific mechanism. The effectiveness of benzodiazepines in IESS overall is not robust though some reports cite clobazam or nitrazepam as a potentially useful agents when IESS fail to respond to standard therapy [82,83]. Surgery is plausible to consider if there is either a focal lesion or area of brain showing focal metabolic abnormality [84]; corpus callosotomy, a palliative procedure, was demonstrated by meta-analysis to eliminate spasms in about one-third of refractory patients [85]. The ketogenic diet has been proposed as an efficacious alternative and even as a first-line agent at some institutions [86,87]. - Cannabidiol is rising in popularity among families but its place in the algorithm is not yet established [88]. Unfortunately, most of these alternative treatments have shown lower effectiveness compared to standard therapies. Standard therapies are effective in at least 50–60 % of children with IESS, compared with less than 10 % effectiveness of non-standard therapies [53]. In summary, treatment of IESS remains complex and controversial, and it is urgent to use animal models to identify alternative therapies.

## Animal Models and Mechanisms

### General considerations

The rarity of IESS and the complexity of human brain development make it challenging to undertake large scale clinical studies addressing pathomechanisms. In designing an animal model of IESS, the typical age of onset, multiplicity of possible etiologies, and aberrant cognitive outcomes must be considered. Presumably, if the initial insult was prenatal or early postnatal (weeks to months prior to IESS onset), there is progressive alteration of brain function that eventually leads to spasms. It is important to appreciate that the brain is undergoing continual and rapid structural and functional development during this interval. Potential sites of pathophysiology could involve ion channels, synaptic connectivity and function, metabolic pathways, and myelination, to name a few. Species-related differences represent one barrier to an exact concordance of animals to human findings yet should not preclude the usefulness of models in addressing fundamental questions.

Existing animal models mirror the etiological diversity of IESS. Whether the etiology is acquired, genetic, or unknown, the goal of animal models is to improve therapy by identifying novel treatments. It is uncertain whether viable personalized medicine will result from this approach. While models can be utilized to screen potential therapeutics, ultimately they should be designed to provide deeper insights into underlying pathogenic mechanisms.

Here I highlight four examples of emerging animal models that might be useful as a step forward in the search of mechanism, with full descriptions of extant animal models available in numerous reviews [6,72,[89], [90], [91], [92], [93], [94], [95], [96], [97]]. Most of these reviews organize the models according to acute vs chronic or acquired vs genetic. Each model meets some of the ideal or sufficient criteria proposed more than two decades ago [98,99]. While each model is valuable for investigating particular pathways or cellular events involved in IESS, no single model should be expected to replicate all phenotypic features of human IESS. It is essential to bear in mind species-specific differences in brain development when comparing the various models and their seizure susceptibility [100]. While it was hoped that animal models would expeditiously pave the way toward novel therapies, translatable results have been limited so far, for many reasons, ranging from species differences to genetic heterogeneity.

Some authors have proposed mechanisms that could lead to IESS. For example, Riikonen lists five categories of potential pathophysiology – excitation/inhibition (E/I) imbalance, alteration of the hypothalamic-pituitary-adrenal axis (HPA) in the setting of a variety of stressors, inflammation, genetic/epigenetic factors, and glucocorticoid effects on brain development [95]. Similarly, Innes and colleagues outline five hypotheses for etiopathogenesis of IESS - genetic/epigenetic factors, stress/HPA activation, neuroinflammation, synaptic dysfunction, and altered metabolic regulation [72]. The overlap is striking between these two lists, but it might be noted that the final common pathway must involve E/I imbalance, so these factors are ultimately hierarchical, all converging on some aspect of E/I balance. If neuronal excitability and seizure predisposition are broadly considered as decreased inhibition, increased excitation, or both, then potential novel therapies can be envisioned using that lens [101].

### Examples - focus on abnormal inhibition

Here I focus on the GABA system, acknowledging that numerous mechanisms likely underlie or modulate IESS. In particular, the role of inflammation in IESS is receiving widespread attention both clinically and experimentally [72,95,102]. Intriguingly, several GABAergic mechanisms and treatments overlap with neuroinflammation in IESS, making this an important area for future investigation.

Neuronal inhibition is mediated by GABA. A variety of genetic pathogenic variants affecting GABAergic function have been identified in epilepsy and in IESS in particular [103,104]. Potential sites of GABA dysfunction include its receptor components and binding sites, synthesis, synaptic release, degradation, reuptake, and interneuron migration and abundance. The following four examples consider potential pathophysiological mechanisms by which GABA dysfunction may be implicated in IESS (Table 3). Other models of IESS, not discussed here, focus on abnormal neuronal excitation, such as the proconvulsant action of CRH as a consequence of increased stress in the developing brain [105], the NMDA model in which glutamate receptors are overactivated [[106], [107], [108]], anti-inflammation and immune suppression [102], variants in genes regulating sodium channel-mediated excitation [109], overactivity of the mTOR pathway [110], severe structural lesions in the multiple-toxin model [111,112], metabolic and microbiome alterations [113, and others; these are considered in the reviews cited above.

**Table 3:** Examples of inhibitory dysfunction in animal models of epileptic spasms.

Model	Pathophysiology	Consequence
TTX	Na channel block → loss IGF-1 → ↓ GABA INs	Epileptic spasms
GABAAR mutation	*GABRB3* variant → ↓ synaptic inhibition	
Down syndrome – Ts65Dn	GABA_B_R overexpression and oversensitivity	
Down syndrome – Ts65Dn	Persistent GABA_A_R-mediated depolarization → ↑ excitation	
Down syndrome – TcMAC21	↑ EPSCs → ↑ excitation ↓ IPSCs → ↓ synaptic inhibition	
Multiple hit	↓ GABAergic parvalbumin interneurons → ↓ synaptic inhibition	

Abbreviations: TTX, tetrodotoxin; Na, sodium; IGF-1, insulin-like growth factor 1; GABA, ɤ-amino-butyric acid; INs, interneurons; GABA_A_R, GABA receptor type A; GABA_B_R, GABA receptor type B; Ts65Dn, mice trisomic for about 2/3 of human Down syndrome; TcMAC21, transchromosomic mouse artificial chromosome 21; EPSCs, excitatory postsynaptic currents; IPSCs, inhibitory postsynaptic currents.

#### Interneuronopathy: neuronal activity suppression and restoration of IGF-1 deficiency

It was hypothesized that chronic suppression of neural activity during specific time windows of brain development could lead to the hyperexcitability inherent in IESS (neuronal desynchronization hypothesis) [114]. Tetrodotoxin (TTX) is a sodium channel blocker that eliminates neural activity. When TTX was infused into the neocortex via an osmotic pump implanted in 10–12 day-old rats, ES-like flexion seizures were observed within 10 days and continued for days to weeks [115]. The existence of a latent period from TTX infusion until spasms occurrence mimics the human condition. EEGs on these animals show large amplitude, low frequency waves followed by electrodecrement, reminiscent of human EEG patterns [35]. The response to ASMs is also similar to human IESS - ACTH eliminated spasms in 66 % of animals in this model and also attenuated the abnormal interictal EEG pattern, while VGB suppressed or delayed the onset of spasms and significantly reduced high frequency oscillations (HFOs), a marker of local excitability [35]. ES in this model originate from layer 5 neocortical pyramidal neurons [116].

Given the similarities with human IESS, the TTX model provides a valuable tool for studying the fundamental neurophysiological basis of IESS related to acquired structural brain abnormalities. Recent work using this model has identified a potential novel therapy. Insulin-like growth factor-1 (IGF-1) is found abundantly throughout the brain and plays a critical role in the maturation of neocortical connectivity, enhances inhibitory interneuron growth, and exerts an anti-inflammatory effect [95,117]; in mice pups, IGF-1 deficiency-related functional and behavioral deficits were rescued by ganaxolone, which enhanced GABAergic tonic inhibition [118].

IGF-1 levels in CSF were found to be low in children with symptomatic IESS and correlated inversely with the degree of cortical damage; those with higher CSF IGF-1 levels responded favorably to ACTH treatment and most in that group fared better cognitively [119]. In another study, children with IESS with hypsarrhythmia had significantly lower serum IGF-1 levels than those without hypsarrhythmia, and ACTH responsiveness varied inversely with IGF-1 level [120]. In children with IESS due to perinatal stroke, IGF-1 levels in surgically resected tissue were decreased [121], leading to the hypothesis that IGF-1 reduction impairs inhibitory neuron synaptogenesis and predisposes to ES. This hypothesis was supported by experiments using IGF-1 knock-out mice, in which IGF-1 levels were restored and ES were abated by administration of IGF-1-derived tripeptide (1–3)IGF-1 [122]. These findings raise the possibility that (1–3)IGF-1 or related therapies could be disease-modifying in IESS, by ameliorating the injury-induced dysmaturation of inhibitory networks [123]. An FDA-approved (1–3)IGF-1 agent, trofinetide, has already shown benefit for treating communication and behavior in children with Rett syndrome and may be amenable for trials in IESS [124]. Moving this therapy forward would require the support of clinical trials by the pharmaceutical industry, and it would be necessary to choose an appropriate study population, i.e., newly diagnosed children with IESS versus those whose IESS has already failed standard therapies (this caveat applies to all novel therapies).

#### GABA-A receptor variant

A large compilation of pathogenic variants defects associated with epilepsy (the Epi4K project) includes children with IESS who were found to have a variant in GABA_A_ receptor genes. One variant associated with IESS is *GABRB3* (c.A328G, p.N110D), which encodes the GABA_A_ receptor β3 subunit. *GABRB3* subserves important functions in neuronal development and seizure predisposition. Using homologous recombination, a heterozygous mutation was created, *Gabrb3*^*+/N110D*^ [125]. Insertion of this mutated gene into mice resulted in a knock-in (KI) line that developed clusters of spasms only during the age window P14-17. The spasms were suppressed by VGB. EEG was performed when the KI mice reached 2–6 months of age, at which time myoclonic and atypical absence seizures occurred, and showed higher amplitude waveforms compared with wild-type littermates, but not hypsarrhythmia *per se*. In addition, KI mice had lower thresholds for seizures elicited by the GABA_A_ receptor antagonist, pentylenetetrazole.

The *Gabrb3*^*+/N110D*^ KI mice displayed impairments on behavioral and psychosocial tests such as decreased mobility in the pre-chamber socialization test (autism-like behaviors), hyperactivity in the open field test (anxiety), and deficits in the Barnes maze (impaired spatial learning and memory). Preliminary investigations into mechanisms by which *Gabrb3*^*+/N110D*^ KI mice exhibit neuronal hyperexcitability, using whole-cell patch clamp recordings, showed decreased GABA_A_ receptor-mediated miniature inhibitory post synaptic currents in neocortical pyramidal neurons, indicating decreased cortical inhibition. Furthermore, multi-unit recordings demonstrated age-related abnormal thalamocortical oscillations with excessive synchrony and hyperexcitability.

Therefore, loss of neuronal GABA_A_ subunit GABRB3-mediated inhibition was associated with increased propensity to ES-like seizures during an early-life developmental window, followed by increased susceptibility to multiple non-spasms seizure types in adulthood. Moreover, these mice displayed impairments in cognition and behavior, hyperexcitability of thalamocortical and neocortical circuits, and abnormal EEG patterns, but not hypsarrhythmia. In addition to VGB, it remains to be determined whether other GABAergic agents exert beneficial effects in GABA_A_ subunit variants; vinpocetine, an alkaloid derived from the periwinkle plant, facilitates GABA currents and was shown to be effective in a patient with a *GABRB3* variant [126].

#### GABA-B receptor overexpression: Down syndrome models

Approximately 5 % of children with Down syndrome (DS, trisomy 21) develop IESS, representing a 100-fold increase over the general population [[127], [128], [129]]. The mechanisms underlying this increased predisposition are unknown [130], but it can be speculated that overexpression of one or more genes on the extra chromosome 21 could be responsible, a hypothesis testable in animal models. A mouse model of DS, called Ts65Dn, has been used for decades to study the pathophysiology of trisomy 21, including ES. In Ts65Dn mice, administration of a GABA_B_ receptor agonist such as gamma-butyrolactone (GBL) leads to extensor spasms associated with ictal spikes and electrodecrements that were abolished by VGB or ACTH [131]. Ts65Dn mice overexpress the G-protein-coupled inward rectifying potassium channel subunit 2 (GIRK2) which increases postsynaptic GABA_B_ currents and facilitates an ES phenotype and EEG changes. Overexpression of GIRK2 is necessary for the production of the IESS-like phenotype [132]. Knockdown of the *Kcnj6* gene (which codes for GIRK2) endowed the mice with resistance to GABA_B_ agonist-induced spasms [133]. However, trisomy of *Kcnj6* is not sufficient for replicating the desired phenotype and the full pathophysiology that underlies spasms-like seizures in this model remains to be determined.

Another factor contributing to excessive excitability in Ts65Dn mice might be depolarizing GABA that persists beyond the usual age at which GABA action switches from excitatory to inhibitory. Excessive or prolonged GABA_A_ receptor excitatory signaling increases neuronal excitability by disrupting Cl^−^ homeostasis and can lead to seizure activity by enhancing GABA-induced depolarization [134], [135]. Treatment of Ts65Dn mice with a GABA_A_ positive allosteric modulator such as diazepam was shown to reduce seizure susceptibility and severity [136].

Despite its contributions to understanding seizures in DS, the Ts65Dn model may be suboptimal because these mice lack numerous genes that are triplicated in human DS and harbor several genes that are not triplicated in human DS. A newer model, transchromosomic mouse artificial chromosome 21 (TcMAC21), carries a copy of human chromosome 21 and therefore harbors a genetic composition very similar to human DS [137]. Exposure of TcMAC21 mice to GBL also results in epileptic spasms and physiological studies have shown that GABA_A_ receptor-mediated inhibitory synaptic transmission is decreased in TcMAC21 cortical neurons compared with euploid neurons [138]. These findings add to evidence supporting a role of abnormal GABAergic inhibition in IESS although the TcMAC21 neurons also express increased sensitivity to kainic acid, adding an increased excitatory component to the spasms physiology (intriguingly, the gene encoding kainic acid receptors resides on chromosome 21) [138,139]. As in Ts65Dn mice, spasms in TcMAC21 mice reflect an ictal phenomenon, occurring only during rhythmic sharp waves on EEG. A limitation of both DS models is the absence of spontaneous spasms. Much more work remains to clarify the details, but this line of research enables mechanistic investigation in IESS models with human chromosomic correlation as well as emphasizing a possible contribution of both inhibitory and excitatory mechanisms [130].

#### Severe structural lesions: inhibitory dysfunction in the multiple-hit model

The mouse multiple-hit model mimics acquired structural (formerly called symptomatic) IESS cases, in which an etiology is known [111]. The model is established by intracerebral injection of three toxins in the first few days of life: the anti-neoplastic agent doxorubicin (DOX) on postnatal day 3, which promotes cytotoxic oxidative damage; intracerebral ventricular administration of the pro-inflammatory compound lipopolysaccharide (LPS) on postnatal day 3, and intraperitoneal injection of the tryptophan hydroxylase inhibitor *p*-chlorophenylalanine (PCPA) on postnatal day 5. DOX and LPS disrupt cortical and subcortical structures and their connections, while PCPA reduces the amount of serotonin in the brain as some cases of IESS exhibit low CSF serotonin metabolites [140]. The resultant structural changes vary somewhat as do structural IESS causes in children, with damage affecting the injected cortical hemisphere plus corpus callosum, striatum, thalamus, and hippocampus. Spasms with IESS-like EEG characteristics are then observed and they often evolve into other seizure types; cognitive deficits and autistic-like behaviors are observed after postnatal day 9 [111].

This model is highlighted here because it has been used extensively for systematic drug testing. ACTH was ineffective but VGB suppressed the spasms, though it was associated with a high mortality rate [85]. To address this issue, the VGB analog CPP-115, which has a higher affinity for GABA aminotransferase and less retinal toxicity, was attempted [141]. Low doses of CPP-115 reduced spasms without increased mortality, but higher concentrations had similar toxicity as VGB. The effectiveness of VGB and its analogs might be explained by the selective reduction of GABAergic cortical parvalbumin interneurons seen in histological studies in this model [142]. Therefore, the multiple-hit model joins those discussed above in implicating a possible effect on inhibitory regulation in IESS.

Other drugs screened using the multiple-hit model include carisbamate, a broad spectrum antiseizure medication thought to block sodium and calcium channels and also exert a modulatory effect on presynaptic GABA receptors with an increase in Cl**^−^** conductance [143]; behavioral and electro-clinical spasms were suppressed [144]. Galanin is a neuroactive steroid that facilitates GABAergic inhibition; galanin agonists have been shown in multiple preclinical models to have antiepileptic and neuroprotective characteristics, but acute injections of NAX 5055 (a galanin analog) did not reduce the number or severity of spasms in the multiple-hit model [145]. Similarly, the caspase 1 inhibitor VX-765 (belnacasan), which counteracts the pro-inflammatory effect of LPS, and the GABA_B_ receptor inhibitor CGP35348 did not have any effect on spasms [146].

Recent attention focused on the effects of drugs that inhibit the nuclear factor kappa like to change enhancer of activated B cells (NF-kB), celastrol and edavarone. These anti-inflammatory, antioxidant drugs were tested in the multiple-hit model [147]. Only celastrol reduced behavioral and electroclinical spasms. Other drugs with anti-inflammatory or immunomodulatory actions (fingolimod, sivelestat) exerted minimal and transient benefit [112]. Likewise, (1–3)IGF-1 had no major or lasting effect on spasms in this model, as opposed to its effectiveness in the TTX model. These results emphasize the benefits of standardized protocols to evaluate potential therapies in IESS models, but also raise the caveat that different IESS models respond differently to a given agent.

Brain insults that were caused by the combination of toxins used to establish this model mimic severe cases of IESS with structural brain damage. In the multiple-hit model, there are several alterations of GABAergic function among many other potential mechanisms. It appears useful for screening proposed anti-IESS compounds, although none of the many tested drugs have yet reached clinical use.

## Concluding remarks

IESS remains an enigmatic epilepsy syndrome with incomplete knowledge about its causes, treatment, and pathophysiology. Consistent terminology will facilitate research and therapeutic trials. Newer diagnostic modalities and enhanced clarity of the EEG features of IESS, especially hypsarrhythmia, will aid the search for novel therapies. Animal models should also help in this respect. While each IESS preclinical model has strengths and drawbacks, in aggregate, the models can be used to better understand this disorder and improve its treatment options. Complexity of the pathogenesis of IESS renders it virtually impossible to replicate all of the features in a single animal model, especially when considering the inherent differences between human and rodent brains. Instead, research should focus on developing targeted strategies to investigate particular molecular or cellular phenomena that are known to contribute to IESS. In defining mechanisms, investigators cannot rely on whether a specific drug works or not to define the mechanism; for example, VGB and (1–3)IGF-1, both of which exert GABAergic effects, work in some models but not others. This does not preclude numerous inhibitory mechanisms from being involved in IESS pathophysiology, as explored here.

New models will undoubtedly be developed, especially those that are related to gene variants, but clarification of the critical knowledge gaps in IESS will require more than just additional models. Each model must be used to rigorously address specific mechanistic questions. The unique as well as overlapping mechanisms of ES suppression versus prevention of epileptogenesis will be critical as the search for new, targetable treatments are sought. This approach will have the highest impact in elucidating the physiological basis of spasms, associated EEG changes, and severe cognitive sequelae of IESS.

## Author Contributions

I am the sole author of this article and therefore my contributions represent 100 %.

## Declaration of competing interest

The author declares that he has no known competing financial interests or personal relationships that could have appeared to influence the work reported in this paper.
